# Effects of tropical forage inclusion in concentrate-based diets on digestibility, growth performance, carcass traits, and meat quality of hair sheep under intensive tropical conditions

**DOI:** 10.14202/vetworld.2026.2252-2263

**Published:** 2026-05-29

**Authors:** David Escalante Raz, Javier Cantón Castillo, Alberto Alcaráz Romero

**Affiliations:** 1 Tecnológico Nacional de México, Campus Conkal, Yucatán, México; 2 Instituto Nacional de Investigaciones Forestales, Agrícolas y Pecuarias (INIFAP), Campo Experimental Mocochá, Yucatán, México

**Keywords:** carcass traits, digestibility, growth performance, hair sheep, intensive feeding, meat quality, tropical forages, tropical sheep production

## Abstract

**Background and Aim:**

Intensive sheep production systems in tropical regions frequently depend on concentrate-based diets to sustain rapid growth and carcass quality; however, increasing grain costs have stimulated interest in incorporating locally available tropical forages as partial substitutes. This study evaluated the effects of including Maralfalfa (*Pennisetum* sp.), Mombaza (*Megathyrsus maximus*), or CT-115 (*Pennisetum purpureum*) in concentrate-based diets on nutrient digestibility, growth performance, carcass traits, and meat quality of hair sheep raised under intensive tropical conditions.

**Materials and Methods:**

Two complementary experiments were conducted at the Mocochá Experimental Field of the National Institute of Forestry, Agricultural, and Livestock Research, Yucatán, México. Apparent digestibility was evaluated in 16 Katahdin × Pelibuey rams (27.2 ± 4.3 kg body weight) assigned to four dietary treatments: concentrate feed alone or concentrate feed supplemented with 30% Maralfalfa, Mombaza, or CT-115 on a dry matter basis. Growth performance, carcass characteristics, and meat quality were assessed in 36 Katahdin × Pelibuey lambs (18.0 ± 3.3 kg body weight) fed the same diets for 84 days. Measurements included dry matter intake, apparent digestibility, average daily gain, slaughter live weight, carcass yield, *Longissimus dorsi* muscle area, pH, drip loss, and proximate composition of meat. Data were analyzed using general linear and mixed-effects models.

**Results:**

Concentrate-fed lambs showed the greatest apparent digestibility of dry matter, crude protein, neutral detergent fiber, and acid detergent fiber, whereas CT-115 reduced nutrient digestibility, particularly fiber utilization (p < 0.01). Nevertheless, forage inclusion significantly increased total dry matter intake compared with concentrate feed alone (p < 0.01). Average daily gain, slaughter live weight, hot carcass weight, carcass yield, backfat thickness, and *Longissimus dorsi* muscle area were not significantly affected by dietary treatment (p > 0.05). Meat pH, temperature, and drip loss remained within normal physiological ranges across all diets. Lambs fed concentrate feed alone exhibited greater intramuscular fat deposition, whereas forage-fed groups showed higher moisture content (p < 0.05).

**Conclusion:**

The inclusion of up to 30% Maralfalfa, Mombaza, or CT-115 in concentrate-based diets maintained growth performance, carcass characteristics, and meat quality in hair sheep under intensive tropical conditions. Although concentrate-only feeding maximized nutrient digestibility, forage supplementation increased feed intake without compromising productive or carcass responses, supporting the nutritional feasibility of tropical forages as partial concentrate substitutes in sheep production systems.

## INTRODUCTION

Sheep farming in tropical regions offers strong development potential due to the adaptability of hair sheep to harsh environmental conditions and their efficiency in utilizing locally available forage. Pasture-based production systems provide both economic and ecological benefits [1], and hair sheep exhibit considerable resilience under heat stress, making them a sustainable livestock option in tropical and subtropical regions [2]. Pelibuey and Blackbelly breeds have traditionally served as maternal genotypes and are frequently crossed with wool breeds as terminal sires, whereas Dorper and Katahdin sheep have more recently been introduced and widely adopted in commercial production systems because of their superior growth potential and carcass performance [3]. Production systems in tropical areas range from extensive grazing systems based on native and introduced grasses, particularly during dry seasons [4], to semi-intensive and intensive confinement systems that rely heavily on grain supplementation and specialized meat breeds or their crossbreeds [5, 6]. Although intensive feeding systems generally improve growth performance and feed efficiency, their profitability remains highly sensitive to fluctuations in grain prices [7]. Because finishing diets commonly contain 80%–90% concentrate feed (CF), rising feed costs have stimulated interest in alternative, locally available feed resources that can partially replace concentrate ingredients without compromising productive performance.

Improved tropical forages have gained attention as potential alternatives in small-ruminant nutrition because of their high biomass production, adaptability to tropical climates, and capacity to provide fiber and nutrients under intensive feeding conditions. Maralfalfa (*Pennisetum* sp.), Mombaza (*Megathyrsus maximus*), and CT-115 (*Pennisetum purpureum*) have been individually reported as productive forages with favorable agronomic characteristics and the potential to support nutrient intake and animal productivity [8–10]. Previous investigations have mainly evaluated these forages under grazing, extensive, or semi-intensive systems, while studies involving single-forage supplementation have produced variable responses depending on forage quality, forage-to-concentrate ratio, and animal genotype. Furthermore, forage inclusion in concentrate-based diets may influence ruminal fermentation, nutrient digestibility, carcass composition, and meat quality attributes, all of which are critical determinants of production efficiency and consumer acceptance.

Despite the increasing interest in tropical forage utilization, important knowledge gaps remain regarding the comparative performance of multiple improved forage species under controlled intensive tropical production systems. Specifically, limited information is available on the simultaneous evaluation of Maralfalfa, Mombaza, and CT-115 as partial replacements for CF in hair sheep diets, particularly regarding apparent digestibility, growth performance, carcass characteristics, and meat quality traits. Most studies have focused on forage agronomic performance or animal growth responses alone, whereas integrated evaluations that combine digestibility, carcass measurements, and meat physicochemical characteristics under intensive tropical conditions remain scarce. In addition, information on the suitability of these forages for Katahdin × Pelibuey lambs raised under confinement conditions remains insufficient.

Therefore, the hypothesis of this study was that partial replacement of CF with improved tropical forages would increase total dry matter intake (DMI) without negatively affecting nutrient digestibility, growth performance, carcass traits, or meat quality of Katahdin × Pelibuey lambs raised under intensive tropical conditions. Accordingly, the objective of this study was to evaluate and compare the effects of including Maralfalfa, Mombaza, or CT-115 in concentrate-based diets on feed intake, apparent digestibility, growth performance, carcass characteristics, and meat composition of hair sheep managed under intensive tropical production systems.

## MATERIALS AND METHODS

### Ethical approval

All experimental procedures involving animals were conducted in accordance with the Mexican Official Standard NOM-062-ZOO-1999 for the care and use of experimental animals [11] and complied with the ARRIVE 2.0 guidelines for reporting animal research [12]. The experimental protocol was reviewed and approved by the Institutional Ethics and Research Committee of the National Institute of Forestry, Agricultural, and Livestock Research (INIFAP) under Approval No. JAG.500-00908. Throughout the experimental period, animal health, behavior, and welfare conditions were monitored daily by trained personnel to minimize stress and ensure appropriate management practices. All handling, housing, feeding, transport, and slaughter procedures were performed in accordance with national animal welfare regulations and internationally accepted ethical standards for livestock experimentation.

### Study period and location

The study was conducted from January to March 2019 at the Mocochá Experimental Field of the INIFAP, located in Yucatán, México (21°09′ N, 89°43′ W; 8 m above sea level). The regional climate is classified as tropical subhumid (Aw) according to the Köppen classification modified by García [13]. The area has a mean annual temperature of 26.5°C and an average annual precipitation of approximately 900 mm, with the rainy season from June to November.

### Study design

The experiment consisted of two complementary trials designed to evaluate the effects of tropical forage inclusion in concentrate-based diets for hair sheep under intensive production conditions. The first trial evaluated apparent *in vivo* digestibility, whereas the second trial assessed growth performance, carcass traits, and meat quality characteristics. In both experiments, animals were assigned to four dietary treatments in a completely randomized design. The treatments consisted of T1 = 100% CF; T2 = 70% CF + 30% Maralfalfa grass (*Pennisetum* sp.); T3 = 70% CF + 30% Mombaza grass (*Megathyrsus maximus*); and T4 = 70% CF + 30% CT-115 grass (*Pennisetum purpureum*) on a dry matter (DM) basis.

### *In vivo* apparent digestibility evaluation

Apparent *in vivo* digestibility was determined as described by Garry *et al*. [14]. Sixteen adult intact Katahdin × Pelibuey rams (27.2 ± 4.3 kg body weight) were individually housed in metabolic cages (1.0 × 0.90 × 0.50 m) equipped with separate feed and water systems and independent feces collection facilities. Animals were randomly assigned to the four dietary treatments, with four replicates per treatment and one animal per cage.

Apparent *in vivo* digestibility (%) was calculated using the following equation:

[(nutrient intake − nutrient in feces)/nutrient intake]×100

### Diets and forage management

Experimental diets were formulated on a DM basis and subsequently adjusted to an as-fed basis before feeding (Tables 1 and 2). CF was offered once daily during the morning, whereas freshly harvested forage was supplied at midday. Maralfalfa, Mombaza, and CT-115 grasses were harvested at their respective regrowth stages of 80, 60, and 80 days, chopped to approximately 4–5 cm, and offered fresh to the animals.

**Table 1:** Ingredient composition and chemical profile of concentrate feed (% Dry matter).

Ingredient	Percentage
Ground sorghum grain	47.32
Canola	11.22
Wheat bran	13.62
Soybean hulls	13.55
Soybean meal	3.85
Cane molasses	5.00
Calcium carbonate	2.80
Common salt	0.80
Urea	0.80
Sodium bicarbonate	0.40
Ammonium sulfate	0.15
Trace minerals^a^	0.43
ADE vitamins	0.06
Total	100.00
Chemical composition	
DM (%)	90.40
Protein (%)	14.80
Calcium (%)	0.65
Phosphorus (%)	0.33
Metabolizable energy (Mcal/kg DM)^b^	2.71

a Provided per kilogram of diet: Mg = 8 mg, Zn = 15 mg, F = 8 mg, Mn = 3 mg, Cu = 10 mg, Se = 0.3 mg, Co = 0.20 mg.

b Based on NRC [17].

**Table 2:** Chemical composition of experimental forages (% Dry matter).

Component (% DM)	Maralfalfa	Mombaza	CT-115
Dry matter	29.60	32.04	28.20
Protein	9.97	8.30	7.61
Neutral detergent fiber	68.96	64.32	73.41
Acid detergent fiber	44.89	47.30	44.72
Calcium	0.48	0.40	0.83
Phosphorus	0.20	0.27	0.17

Before the experiment began, sheep were dewormed with ivermectin (Ivermectin®, Laboratorios Sanfer, México City, México) and adapted to the diets and housing conditions for 14 days prior to data collection.

### Sample collection and laboratory analyses

The digestibility trial lasted 7 days, during which daily feed intake and fecal output were recorded. Composite samples consisting of 10% of the daily feces, feed offered, and feed refusals were collected, pooled, dried at 65°C for 48 h, and stored at −20°C until analysis.

DM (AOAC 950.46), crude protein (CP; AOAC 981.10; N × 6.25), and ash (AOAC 920.153) were determined using standard AOAC methods [15]. Neutral detergent fiber (NDF) and acid detergent fiber (ADF) were analyzed following the procedures described by Van Soest *et al*. [16].

### Growth performance evaluation

Thirty-six intact Katahdin × Pelibuey male lambs (18.0 ± 3.3 kg initial body weight) were stratified according to body weight and assigned to the four dietary treatments (n = 9 per treatment) in a completely randomized design. Diets were formulated according to NRC [17] recommendations for growing lambs to achieve an average daily gain (ADG) of 200–250 g/day.

Animals were individually housed in shaded pens (3.0 × 1.6 m) with *ad libitum* access to feed and fresh water. Following deworming and a 14-day adaptation period, lambs were weighed after a 16 h fasting period at the beginning of the experiment and subsequently every 14 days throughout the 84-day feeding period. Final body weight was recorded at the end of the experiment, and ADG was calculated as the difference between final and initial body weight divided by the number of experimental days.

### Slaughter procedures and carcass evaluation

At the end of the feeding trial, lambs were transported to a commercial slaughter facility, fasted for 16 h, and slaughtered in accordance with NOM-033-SAG/ZOO regulations for humane slaughter [18]. Slaughter live weight (SLW) and hot carcass weight (HCW) were recorded to calculate carcass yield (CY) using the following equation:

CY (%) = (HCW/SLW) × 100

After chilling for 24 h at 4°C, carcasses were sectioned between the 12th and 13th ribs to measure backfat thickness (BF) using a stainless-steel ruler, as described by Elizalde *et al*. [19]. The *Longissimus dorsi* muscle (LDM) area was determined using a transparent grid following the Ohio State University protocol [20].

### Meat physical quality assessment

After 24 h of chilling, the LDM was exposed between the 12^th^ and 13^th^ ribs. Muscle pH was measured using a portable pH meter (HI99163 Meat pH Meter, Hanna Instruments, Woonsocket, RI, USA) previously calibrated with standard buffer solutions at pH 4.0 and 7.0. Measurements were obtained by inserting the probe approximately 2 cm into the geometric center of the muscle.

Immediately after pH determination, muscle temperature was measured at the same location using a digital thermometer (Taylor Precision Products Inc., Oak Brook, IL, USA).

The LDM was subsequently excised, and 2.5-cm-thick steaks were collected for the determination of drip loss (DL) at 24 and 48 h postmortem, as described by Lunesu *et al*. [21]. Three replicate samples (100 g each) from each carcass were placed in polyethylene bags, suspended in a refrigerated chamber at 4°C, and reweighed after each storage period. DL was expressed as the percentage of weight loss during storage. Outliers exceeding ±1.5 interquartile ranges were identified and excluded before statistical analysis.

DL (%) was calculated as:


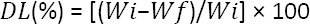


where *W i* represents the initial sample weight (g), and *W f* represents the final sample weight after drainage (g).

### Proximate and chemical composition of meat

For proximate analysis, 150 g samples of LDM were vacuum-packed, stored at −20°C, thawed at 4°C, trimmed of visible fat, minced, and homogenized for 15 s. Moisture (AOAC 950.46), ash (AOAC 920.153), CP by the Kjeldahl method (AOAC 981.10; N × 6.25), and intramuscular fat by Soxhlet extraction (AOAC 991.36) were analyzed according to AOAC methods [15].

All analyses were performed in technical duplicate using a Kjeldahl system (Kjeltec™ 8200, FOSS, Hillerød, Denmark) and a Soxhlet extraction system (Thermo Scientific™ SE400, Thermo Fisher Scientific, Waltham, MA, USA).

### Statistical design and data analysis

Data obtained for apparent *in vivo* digestibility, growth performance, carcass traits, chemical composition, pH, and temperature were analyzed using the following general linear model:


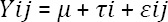


where *Yij*represents the response variable of the j-th animal receiving the i-th dietary treatment, *μ* is the overall mean, *𝜏i* is the fixed effect of dietary treatment, and *𝜀ij* is the residual error.

Before analysis, data normality was evaluated using the Shapiro–Wilk test, whereas homogeneity of variances was assessed using Levene’s test. When significant treatment effects were detected, means were separated using Tukey’s honestly significant difference test to control the overall Type I error rate.

Statistical analyses were performed using SAS software version 9.1 (SAS Institute Inc., Cary, NC, USA) [22]. Results are presented as mean ± standard error. Statistical significance was declared at p < 0.05.

A post hoc power analysis (1 − β = 0.80; α = 0.05) indicated that the experimental design was sufficient to detect minimum differences of approximately 15% for digestibility-related variables. Experimental data were recorded daily using standardized forms and subsequently entered into electronic spreadsheets. Data entries were independently verified, and no missing values were identified.

DL data were analyzed using a mixed-effects model to account for repeated measurements within carcasses. The statistical model used was:


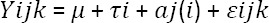


where *Yijk* represents the DL value of the k-th replicate from the j-th animal receiving the i-th dietary treatment, *μ* is the overall mean, *𝜏i* is the fixed effect of diet, *aj*(*i*) is the random effect of animal nested within treatment, and *𝜀ijk* is the residual error.

Time points (24 and 48 h postmortem) were considered repeated measures within animals, and the covariance structure was selected according to model fit criteria. Analyses were performed using PROC MIXED in SAS software version 9.1 [22]. Least squares means were compared using Tukey’s test, and significance was declared at p < 0.05.

## RESULTS

### Diet digestibility

Dietary effects on feed intake and nutrient digestibility are presented in Table 3. Significant differences in concentrate intake were observed among treatments (p < 0.05). Lambs fed the concentrate-only diet (CF) showed the highest concentrate intake, which did not differ significantly from CF + Maralfalfa or CF + CT-115, but was significantly greater than CF + Mombaza. Regarding forage intake, lambs receiving CF + Mombaza exhibited the highest forage consumption (p < 0.01), followed by CF + Maralfalfa and CF + CT-115.

**Table 3:** Effect of forage inclusion on feed intake and nutrient digestibility in lambs.

Variables	T1	T2	T3	T4	p-value	Standard error
Feed intake (kg DM/animal/day)						
Concentrate feed	1.151ᵃ	1.105ᵃᵇ	0.978ᵇ	1.108ᵃᵇ	0.039	0.2160
Forage	0.000ᶜ	0.293ᵇ	0.377ᵃ	0.276ᵇ	0.001	0.0600
Total DM	1.151ᵇ	1.399ᵃ	1.354ᵃ	1.385ᵃ	0.001	0.2320
Digestibility (%)						
DM	82.226ᵃ	79.921ᵃᵇ	78.851ᵃᵇ	78.170ᵇ	0.014	4.2207
CP	80.283ᵃ	79.745ᵃᵇ	78.238ᵃᵇ	78.040ᵇ	0.008	4.2865
Ash	82.318ᵃ	79.828ᵃᵇ	78.864ᵇ	78.423ᵇ	0.018	4.2558
NDF	81.915ᵃ	80.031ᵃᵇ	78.910ᵃᵇ	78.160ᵇ	0.015	4.2207
ADF	82.131ᵃ	79.211ᵃᵇ	78.665ᵇᶜ	76.422ᶜ	0.001	4.1369

T1 = Concentrate feed (CF), T2 = CF + Maralfalfa grass, T3 = CF + Mombaza grass, T4 = CF + CT-115 grass.

^a–c^ Values within a row with different superscripts differ significantly (p < 0.05).

Total DMI was significantly greater (p < 0.01) in all forage-supplemented diets compared with the CF diet, indicating that forage inclusion increased overall nutrient intake without reducing total feed consumption. Significant differences (p < 0.01) were also observed for all digestibility variables. Lambs fed the CF diet showed the greatest digestibility coefficients for DM, CP, ash, NDF, and ADF. Diets containing CF + Maralfalfa or CF + Mombaza presented intermediate digestibility values. Acid detergent fiber digestibility in the CF + Maralfalfa treatment was statistically similar to that of CF, whereas CF + Mombaza exhibited lower ADF digestibility than CF (p < 0.01). The lowest digestibility coefficients were consistently observed in lambs fed CF + CT-115, particularly for ADF digestibility (p < 0.01), highlighting the influence of forage source on nutrient utilization.

### Growth performance and carcass traits

Growth performance and carcass characteristics are presented in Table 4. No significant differences were detected among treatments for growth performance or carcass variables (p > 0.05). ADG ranged from 0.240 to 0.265 kg/day, whereas SLW varied from 43.50 to 44.87 kg. Likewise, HCW ranged from 21.09 to 21.82 kg, and CY varied from 48.32% to 48.61%.

**Table 4:** Effect of forage inclusion on growth performance and carcass traits in lambs.

Variables	T1	T2	T3	T4	p-value	Standard error
SLW (kg)	44.871	43.500	43.676	43.767	0.5608	2.2343
ADG (kg/day)	0.240	0.265	0.256	0.256	0.769	0.0500
HCW (kg)	21.822	21.088	21.111	21.244	0.693	1.4785
CY (%)	48.56	48.45	48.32	48.61	0.990	1.998
BF (mm)	1.111	1.100	0.088	0.083	0.591	0.0460
LDM area (cm²)	14.659	14.372	15.520	14.982	0.702	2.1439

T1 = Concentrate feed (CF), T2 = CF + Maralfalfa grass, T3 = CF + Mombaza grass, T4 = CF + CT-115 grass.

Backfat thickness values were similar among treatments (p > 0.05), with measurements close to 1.1 mm in CF, CF + Maralfalfa, and CF + Mombaza treatments, whereas slightly lower values were observed in the CF + CT-115 group. The largest LDM area was recorded in lambs fed CF + Mombaza, whereas the smallest area was observed in the CF + Maralfalfa treatment; however, these differences were not statistically significant (p > 0.05).

### Meat physical characteristics

**Figure 1:**
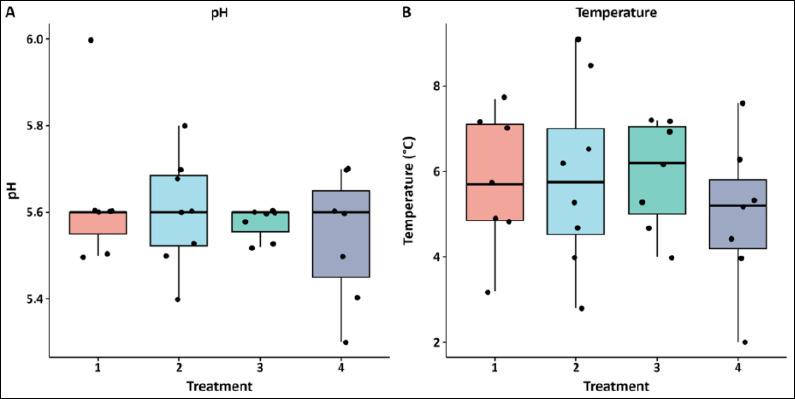
Distribution of (A) pH and (B) temperature at 24 h postmortem in the *Longissimus dorsi* muscle of lambs fed different dietary treatments: T1 = concentrate feed (CF), T2 = CF + Maralfalfa grass, T3 = CF + Mombaza grass, and T4 = CF + CT-115.

Meat physical traits did not differ significantly among treatments (p ≥ 0.05); therefore, descriptive statistics were used for reporting (Table 5). Median ultimate pH values at 24 h postmortem ranged from 5.5 to 5.8 and were comparable across diets, with lower variability in CF and CF + Mombaza and slightly greater dispersion in CF + Maralfalfa and CF + CT-115 (Figure 1A). Meat temperature during chilling ranged from 5.0°C to 6.2°C, with CF + CT-115 showing the lowest median and CF + Mombaza the highest median (Figure 1B).

**Table 5:** Descriptive statistics for pH, temperature, and drip loss of *Longissimus dorsi* muscle.

Attributes	Median	Standard error	Minimum	Maximum	Coefficient of variation (%)
pH	5.587	0.024	5.300	6.000	2.29
Temperature (°C)	5.651	0.316	2.000	9.100	30.13
DL (24 h)	0.9975	0.044	0.300	1.730	37.68
DL (48 h)	1.5708	0.065	0.638	2.687	35.11

**Figure 2:**
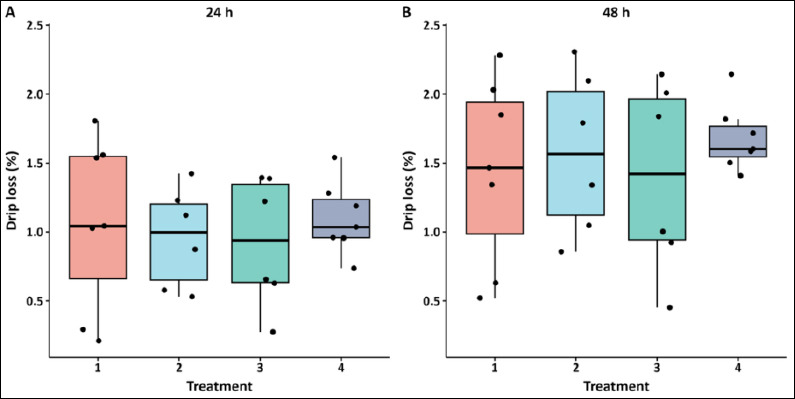
Distribution of drip loss in the *Longissimus dorsi* muscle at (A) 24 h and (B) 48 h postmortem in lambs fed different dietary treatments: T1 = concentrate feed (CF), T2 = CF + Maralfalfa grass, T3 = CF + Mombaza grass, and T4 = CF + CT-115.

DL medians at 24 h ranged from 0.85% to 1.10%. The CF + Mombaza group showed the widest variability, ranging from 0.5% to 1.5%, whereas CF displayed the narrowest interquartile distribution, despite the presence of a low outlier. CF + Maralfalfa and CF + CT-115 exhibited intermediate ranges, with slightly higher medians than CF and CF + Mombaza (Figure 2A).

At 48 h, DL medians increased to 1.2%–1.8%. CF showed the broadest variability, ranging from 0.6% to 2.6%, whereas CF + Mombaza had the narrowest range, from 1.0% to 2.0%. Similar to the 24 h results, CF + Maralfalfa and CF + CT-115 presented intermediate variability, with comparable medians across treatments (Figure 2B). Although the range of values was wide, no statistically significant differences were detected among treatments (p > 0.05).

### Meat chemical composition

The chemical composition of lamb meat is presented in Table 6. Moisture content was significantly greater (p < 0.05) in lambs fed CF + Mombaza compared with those fed the CF diet, whereas intermediate values were observed in CF + Maralfalfa and CF + CT-115 treatments.

**Table 6:** Effect of forage inclusion on proximate composition of lamb meat.

Variables	T1	T2	T3	T4	p-value	Standard error
Moisture (%)	70.31ᵃ	71.99ᵃᵇ	72.74ᵇ	71.00ᵃᵇ	0.025	1.717
CP (% DM)	19.89	20.99	20.78	20.41	0.081	0.920
Ash (% DM)	0.99	1.03	1.03	1.01	0.441	0.055
Total fat (% DM)	7.93ᵃ	4.65ᵇ	4.92ᵃᵇ	5.58ᵃᵇ	0.016	2.150

T1 = Concentrate feed (CF), T2 = CF + Maralfalfa grass, T3 = CF + Mombaza grass, T4 = CF + CT-115 grass.

ᵃ,ᵇValues within a row with different superscripts differ significantly (p < 0.05).

Crude protein and ash contents did not differ significantly among treatments (p > 0.05), with mean values of approximately 20.52% and 1.02% on a DM basis, respectively. Total fat content was highest in lambs fed the CF diet and lowest in those receiving CF + Maralfalfa (p < 0.05). Intermediate fat values were observed in CF + Mombaza and CF + CT-115 treatments.

## DISCUSSION

### Nutrient digestibility of diets

The inclusion of both CF and forage in sheep diets has been widely reported to enhance ruminal function and improve nutrient utilization, often resulting in greater DMI. This trend was partially observed in the present study, where forage-based diets stimulated feed intake. Similar findings have been reported by Obeidat *et al*. [23] and Liu *et al*. [24], who demonstrated that varying forage proportions in total mixed rations stabilize rumen fermentation and support higher intake in lambs. Mohammed *et al*. [25] also reported comparable DMI in lambs fed Mombaza hay versus other forages. Conversely, reduced DMI has been reported when less digestible or more fibrous forages, such as Maralfalfa, are used [26]. This finding was recently supported by de Azevedo *et al*. [27], who showed that forage intake increases as forage availability and quality improve, underscoring the direct role of these factors in determining consumption in small ruminants. Collectively, these findings indicate that DMI responses are shaped by forage characteristics, the animal’s physiological status, and the overall dietary profile.

Nutrient digestibility in the present experiment ranged from 76% to 80%, values considered high for forage-based diets and likely reflecting CF’s contribution to the ration. The lower ADF digestibility observed in lambs fed CT-115 compared with those fed Maralfalfa may be explained by the higher NDF and ADF contents of CT-115, which indicate a greater proportion of structural carbohydrates and potential lignification. These factors are known to limit ruminal fiber degradation and reduce apparent digestibility. However, the increased feed intake observed in forage-based diets may partially offset lower digestibility, resulting in sufficient nutrient availability to sustain growth despite potential reductions in feed efficiency.

These findings are consistent with Muhammad *et al*. [28], who reported improved digestibility with increasing concentrate inclusion, but contrast with the lower digestibility values of 51%–69% reported by Partida *et al*. [26] and Ibrahim *et al*. [29]. Overall, the results support the general agreement that increasing the proportion of forage, particularly with more fibrous forages, reduces DM and organic matter digestibility [30].

### Growth performance and carcass characteristics

The ADG observed in this study was slightly higher than previously reported for Pelibuey lambs. Previous studies reported ADG values of 195–205 g/day in animals fed diets containing agricultural by-products, crop residues, or fresh Mombaza grass, supplemented with concentrate [31, 32]. The improved performance observed in the present study may also be attributed to genotype effects, as Katahdin crosses are recognized for greater growth potential than Pelibuey and Blackbelly lambs [33, 34]. The lower ADG reported by Mohammed *et al*. [25] in older sheep further supports the role of age and physiological status in modulating growth performance.

No significant differences were observed in SLW, HCW, CY, or BF thickness among treatments. These findings support the concept that carcass quality is more closely related to slaughter weight than to diet composition or forage type when nutrient supply is adequate. Wang *et al*. [35] reported minimal effects of the feeding system on lamb carcass parameters. Guim *et al*. [36] also observed negligible effects of different roughage-to-concentrate ratios on carcass traits in Santa Inês lambs. The similar slaughter weights across treatments in the present study likely explain the absence of significant differences in carcass characteristics.

### Meat physical characteristics

Meat quality parameters were within normal physiological ranges. Ultimate pH values of 5.5–5.8 confirmed adequate postmortem glycolysis and the absence of pale, soft, exudative meat or dark, firm, dry meat defects. This finding is consistent with Holman *et al*. [37] and Ge *et al*. [38], who reported similar pH declines in lamb longissimus muscles during rigor mortis. Meat temperature during chilling showed some variation among carcasses but did not differ among dietary treatments. This finding agrees with Muela *et al*. [39], who demonstrated that conventional chilling effectively maintains appropriate temperature and pH dynamics regardless of feeding regimen.

DL at 24 and 48 h postmortem was not significantly affected by diet (p > 0.05). At 24 h, CF-fed lambs showed the least variability, whereas at 48 h they displayed a wider range of values, reflecting normal temporal changes in muscle structure. The more uniform DL at 24 h may be related to consistent early pH decline, while greater variation at 48 h corresponds to progressive alterations in myofibrillar proteins and weakening of the extracellular network during storage [40, 41]. In contrast, CF + Mombaza consistently showed narrower ranges at both time points, suggesting a more stable water-holding capacity. These observations agree with Orzuna *et al*. [42], who reported similar variability patterns at 24 h. Liu *et al*. [43] also demonstrated that diet composition and forage inclusion can modulate the dynamics of water-holding capacity during postmortem aging. Overall, these findings indicate that although dietary treatments did not significantly alter DL, forage inclusion, particularly Mombaza, may contribute to greater stability of water-holding capacity over time.

It should be noted that ruminal fermentation parameters and methane emissions were not measured in this study. Therefore, the observed effects of forage inclusion on intake, digestibility, and meat quality should be interpreted based on productive and carcass responses rather than direct rumen metabolic indicators.

### Meat chemical composition

Chemical composition analysis showed higher intramuscular fat and lower moisture in CF-fed lambs, consistent with the greater energy density of cereal- and oilseed-based diets that promote lipid deposition at the expense of water content [44, 45]. In contrast, forage-based treatments produced leaner carcasses with higher moisture, likely due to their higher fiber and lower energy content, which favor protein accretion and greater water retention [46]. These results emphasize the influence of diet composition on meat characteristics, with practical implications for eating quality. Higher intramuscular fat is associated with improved juiciness and flavor [47], whereas leaner meat with greater moisture content aligns with consumer preferences for healthier, lower-fat products [48].

Although productive performance, carcass traits, and meat quality were evaluated, rumen fermentation parameters and methane emissions were not measured in the present study and should be addressed in future research.

## CONCLUSION

The inclusion of improved tropical forages in concentrate-based diets successfully maintained growth performance, carcass traits, and meat quality of Katahdin × Pelibuey lambs raised under intensive tropical conditions. Diets containing up to 30% Maralfalfa, Mombaza, or CT-115 increased total DMI while preserving ADG, SLW, HCW, CY, and overall carcass quality comparable to those obtained with the concentrate-only diet. Although the CF diet produced the highest nutrient digestibility values for DM, CP, NDF, and ADF, forage inclusion did not negatively affect productive performance. Among the evaluated forages, CT-115 showed comparatively lower fiber digestibility, whereas Mombaza supplementation promoted greater forage intake and more stable DL responses during postmortem storage. Meat pH, temperature, and DL remained within normal physiological ranges across all treatments, confirming that forage supplementation did not compromise the physical quality of the meat. In addition, forage-based diets produced leaner meat with lower intramuscular fat and higher moisture content, characteristics that may be attractive for health-conscious consumers.

From a practical perspective, the results demonstrate that improved tropical forages can partially replace CF in intensive sheep feeding systems without impairing animal performance or carcass value. This finding is particularly relevant for tropical production systems where feed costs and grain price fluctuations substantially affect profitability. The use of locally available forage resources such as Maralfalfa, Mombaza, and CT-115 may help reduce dependence on concentrate ingredients while supporting more sustainable and economically resilient sheep production systems under tropical conditions.

One of the major strengths of this study was the integrated evaluation of nutrient digestibility, growth performance, carcass characteristics, and meat quality within the same experimental framework. In addition, the simultaneous comparison of three improved tropical forages under intensive confinement conditions provides valuable information for practical feeding strategies in hair sheep production systems, an area that remains insufficiently investigated in tropical environments.

However, some limitations should be acknowledged. Ruminal fermentation characteristics, methane emissions, microbial dynamics, and economic cost-benefit analyses were not evaluated. Furthermore, the study focused on a single forage inclusion level and one crossbred genotype in a specific tropical environment, which may limit the broader extrapolation of the results to other production systems or environmental conditions.

Future research should therefore investigate different forage inclusion rates, long-term feeding effects, ruminal fermentation profiles, methane production, nutrient-use efficiency, and economic feasibility under commercial conditions. Additional studies evaluating fatty acid composition, sensory characteristics, and consumer acceptance of meat from forage-supplemented diets would also provide valuable information to optimize tropical sheep production systems.

Overall, the present findings support the nutritional feasibility of partially replacing CF with improved tropical forages in intensive hair sheep production systems. Such feeding strategies may improve sustainability and reduce feeding costs while maintaining satisfactory productive performance, carcass traits, and meat quality under tropical conditions.

## DATA AVAILABILITY

Supplementary data are available from the corresponding author upon request.

## AUTHORS’ CONTRIBUTIONS

JC: Conceptualization and study design. DE: Conducted the animal experiments. AA: Performed the statistical analyses and interpreted the data. JC and AA: Drafted and revised the manuscript. All authors read and approved the final version of the manuscript.

## References

[1] Poli CHE, Monteiro ALG, Devincenzi T, Albuquerque FHM, Motta JH, Borges LI, Muir JP (2020). Management strategies for lamb production on pasture-based systems in subtropical regions: A review. Front Vet Sci.

[2] Oyieng E, Ojango JMK, Gauly M, Ekine-Dzivenu CC, Mrode R, Clark EL, Oloo R, Konig S (2025). The impact of heat stress on growth and resilience phenotypes of sheep raised in a semi-arid environment of sub-Saharan Africa. Livest Sci.

[3] Chay CJ, García HR, Magaña MJ, Macías CU, Luna PC (2019). Productivity of Pelibuey and Katahdin ewes in the humid tropics. Ecosist Recur Agropec.

[4] Moyo B, Ravhuhali KE (2023). Dry season feeding strategies and winter forage production by communal area sheep farmers of the Eastern Cape province in South Africa. Cogent Food Agric.

[5] Moorby JM, Fraser MD (2021). Review: New feeds and new feeding systems in intensive and semi-intensive forage-fed ruminant livestock systems. Animal.

[6] Salinas-Martínez JA, Posadas-Domínguez RR, Ángeles-Hernández JC, Morales-Díaz LD, Rebollar-Rebollar S, Rojo-Rubio R, Arriaga-Jordán CM (2022). The economic effects of grazing in small-scale lamb fattening production systems in central México through a scenario analysis. Trop Anim Health Prod.

[7] Young M, Kingwell R, Young J, Vercoe V (2020). An economic analysis of sheep flock structures for mixed enterprise Australian farm businesses. Aust J Agric Res Econ.

[8] Euclides BV, do Valle BC, Motta MM, de Almeida GR, Montagner BD, Amorim BR (2010). Brazilian scientific progress in pasture research during the first decade of XXI century. Rev Bras Zootec.

[9] Maldonado QH, Carrete CF, Reyes EO, Sánchez AJ, Murillo OM, Araiza RE (2021). Yield and nutritional value of Maralfalfa grass (*Pennisetum* sp.). at different ages. Rev Fitotec Mex.

[10] Esperanza de Dios LG, Ramos JJ, Izquierdo RF, Joaquín TB, Meléndez NF (2022). Productive performance and nutritional value of *Pennisetum purpureum* cv. Cuba CT-115 grass at different regrowth ages. Rev Mex Cienc Pecu.

[11] NOM-062-ZOO (1999). Mexican Official Standard (Norma Oficial Mexicana, NOM):Technical specifications for the production, care, and use of laboratory animals.

[12] Percie du Sert N, Hurst V, Ahluwalia A, Alam S, Avey MT, Baker M, Würbel H (2020). The ARRIVE guidelines 2.0: Updated guidelines for reporting animal research. PLoS Biol.

[13] García E (2004). Modification to the Köppen climate classification system. Geografic Institute. UNAM, México.

[14] Garry B, McGovern FM, Boland TM, Rinne M, Kuoppala K, Baumont R, Lewis E, O'Donovan M (2021). How does herbage mass affect voluntary dry matter intake and *in vivo* organic matter digestibility in sheep and the *in vitro* gas production of perennial ryegrass?. Livest Sci.

[15] AOAC International (2005). Official methods of analysis.

[16] Van Soest PV, Robertson JB, Lewis BA (1991). Methods for dietary fiber, neutral detergent fiber, and nonstarch polysaccharides in relation to animal nutrition. J Dairy Sci.

[17] NRC (2007). Sheep, goats, cervids, and New World camelids.

[18] NOM-033-SAG/ZOO (2014). Official Mexican Standard (Norma Oficial Mexicana, NOM):Methods for the humane slaughter of domestic and wild animals.

[19] Elizalde F, Hepp C, Reyes C, Tapia M, Lira R, Morales R, Sales F, Catrileo A, Silva M (2020). Growth, carcass and meat characteristics of grass-fed lambs weaned from extensive rangeland and grazed on permanent pastures or alfalfa. Animals.

[20] The Ohio State University Extension (2011). 194R sheep resource handbook. Changes in the 2011 edition. Ohio.

[21] Lunesu MF, Battacone G, Mellino MR, Carta S, Pulina G, Nudda A (2023). The heavy suckling lamb of Sarda dairy sheep and its crossbreed with Dorper rams: Performance, meat quality and consumer perceptions. Meat Sci.

[22] SAS Institute Inc (2003). Statistical analysis software SAS/STAT®. User's guide. Version 9.1.

[23] Obeidat BS, Subih HS, Ata M (2020). Protein supplementation improves performance of lambs fed low-quality forage. Animals.

[24] Liu M, Wang Z, Sun L, Wang Y, Li J, Ge G, Jia Y, Du S (2023). Effects of different forage proportions in fermented total mixed ration on muscle fatty acid profile and rumen microbiota in lambs. Front Microbiol.

[25] Mohammed HF, Husam HN, Ahmed AA (2021). The effect of using *Panicum* Mombasa hay and millet hay in the diet on the production performance of Awassi lambs. Med Legal Update.

[26] Partida HM, Loya OJ, Gómez GA, Ramírez RJ, Hernández BJ, Amezcua JT, Escalera VF (2019). Replacement of sorghum grain with *Guazuma ulmifolia* fruit in lamb diets with different forages. Ecosist Recur Agropec.

[27] de Azevedo EB, Savian JV, do Amaral GA, de David DB, Gere JI, Kohmann MM, Bremm C, Jochims F, Zubieta AS, Gonda HL, Bayer C, de Faccio Carvalho PC (2021). Feed intake, methane yield, and efficiency of utilization of energy and nitrogen by sheep fed tropical grasses. Trop Anim Health Prod.

[28] Muhammad FA, Muhammad AR, Karamo J, Sajjad K, Muhammad R, Qamar B, Urooj A, Sibtain A, Hassan MB, Fahd R (2022). Effect of forage-to-concentrate ratio on growth performance and feeding behavior of Thalli lambs. Trop Anim Health Prod.

[29] Ibrahim MK, Adel MA, Uchema YA, Sobhy MS, Hani ME (2021). Comparative digestibility and rumen fermentation of camels and sheep fed different forage sources. Anim Biotechnol.

[30] Na Y, Hua Li D, Lee S (2017). Effects of dietary forage-to-concentrate ratio on nutrient digestibility and enteric methane production in growing goats (*Capra hircus hircus*) and Sika deer (*Cervus nippon hortulorum*). Asian-Australas J Anim Sci.

[31] Macedo R, Aguilar LA (2005). Productive performance of Pelibuey lambs fed a diet based on agro-industrial by-products and crop residues. Livest Res Rural Dev.

[32] Díaz EV, Sánchez RG, Ojeda HM, Casanova LF, Oros OI, Trejo OA, Santos HR (2017). Comparison of productive and financial parameters of hair sheep fed in silvopastoral systems based on *Leucaena leucocephala* and confinement. XLIV Cientific Meeting. AMPA. UNACH-AMPAA.

[33] Macías CU, Álvarez VF, Rodríguez GJ, Correa CA, Torrentera ON, Molina RL, Avendaño RL (2010). Growth and carcass traits of purebred Pelibuey and F1 crossbred lambs with Dorper and Katahdin breeds under confinement conditions. Arch Med Vet.

[34] Vázquez SM, Partida DA, Rubio LM, Medina MD (2011). Productive behavior and carcass characteristics of lambs from the crossbreeding of Katahdin ewes with males from four specialized meat breeds. Rev Mex Cienc Pecu.

[35] Wang W, Zhang X, Wei H, Wang S, Ye Y, He L, Zhang K, Lu Y, Zhang Z, Huang Y (2024). Effects of feeding systems on the growth performance, carcass characteristics, and meat quality in sheep: A meta-analysis. Animals.

[36] Guim A, de Souza SK, Veraz R, Barbosa PM, Félix de Abreu K, Cardoso DB (2023). Carcass characteristics and meat quality of lambs fed diets with different roughage: concentrate ratios supplemented with liquid residue of cassava. Acta Sci Anim Sci.

[37] Holman BW, Kerr MJ, Refshauge G, Diffey SM, Hayes RH, Newell MT, Hopkins DL (2021). Postmortem pH decline in lamb semitendinosus muscle and its relationship to the pH decline parameters of the *longissimus lumborum* muscle: A pilot study. Meat Sci.

[38] Ge Y, Zhang D, Zhang H, Li X, Fang F, Liang C, Wang Z (2021). Effect of postmortem phases on lamb meat quality: A physicochemical, microstructural and water mobility approach. Food Sci Anim Resour.

[39] Muela E, Sañudo C, Campo MM, Medel I, Beltrán JA (2010). Effects of cooling temperature and hot carcass weight on the quality of lamb. Meat Sci.

[40] Huff LE, Lonergan SM (2005). Mechanisms of water-holding capacity of meat: The role of postmortem biochemical and structural changes. Meat Sci.

[41] Warner RD (2017). The eating quality of meat—IV water-holding capacity and juiciness. In:Lawrie's Meat Science.

[42] Orzuna OJ, Dorantes IG, Lara BA, Mendoza MG, Miranda RL, López OR, Hernández GP (2021). Productive performance, carcass traits, and meat quality in finishing lambs supplemented with a polyherbal mixture. Agriculture.

[43] Liu M, Wang Z, Sun L, Wang Y, Li J, Ge G, Jia Y, Du S (2023). Effects of different forage proportions in fermented total mixed ration on muscle fatty acid profile and rumen microbiota in lambs. Front Microbiol.

[44] Pethick D, Harper GS, Oddy VH (2004). Growth, development and nutritional manipulation of marbling in cattle: A review. Aust J Exp Agric.

[45] Hocquette JF, Gondret F, Baeza E, Médale F, Jurie C, Pethick DW (2010). Intramuscular fat content in meat-producing animals: Development, genetic and nutritional control, and identification of putative markers. Animal.

[46] Lee JH, Wildeus S, O'Brien D, Kouakou B (2024). Impact of agro-byproduct supplementation on carcass traits and meat quality of hair sheep and wool ×hair crossbreds grazing on fescue pasture. Animals.

[47] Al-Ghamdi S, Al-Baadani HH, Soufan W, Suliman GM, Abdelrahman MM, Alhidary IA (2024). Influence of varied sprouted barley feeding levels on carcass traits, meat quality and fatty acid profile of lambs. Cogent Food Agric.

[48] Queiroz LO, Barbosa AM, Mourão GB, Fonseca MA, Pinto LF, da Silva J, Silva TM, Lima AG, Bezerra LR, Oliveira RL (2021). Performance, carcass traits and meat quality of lambs fed with different roughage: concentrate ratios associated with variable physically effective neutral detergent fiber content. J Agric Sci.

